# Challenges in high-sensitive troponin assay interpretation for intensive therapy

**DOI:** 10.5935/0103-507X.20190001

**Published:** 2019

**Authors:** Humberto Andres Vaz, Raphael Boesche Guimaraes, Oscar Dutra

**Affiliations:** 1 Unidade de Terapia Intensiva, Instituto de Cardiologia do Rio Grande do Sul, Fundação Universitária de Cardiologia - Porto Alegre (RS), Brasil.

**Keywords:** Troponin T, Troponin I, Myocardial infarction, Biomarkers, Sepsis, Intensive care units

## Abstract

Cardiac troponins T and I are considered highly sensitive and specific markers for the diagnosis of acute myocardial infarction. Currently, a series of nonprimary cardiac abnormalities may manifest as an elevation in high-sensitive assays. The reduction in their detection limits has allowed earlier diagnosis and the use of evidence-based therapeutic measures; however, this characteristic has increased the spectrum of detectable noncoronary heart diseases, which poses challenges for characterizing acute coronary syndromes and creates a new role for these tests in known disorders in intensive care units, especially sepsis. Management of patients through a greater understanding of how these markers behave should be re-evaluated to ensure their correct interpretation.

## INTRODUCTION

Biochemical analysis of myocardial necrosis markers, especially cardiac troponins (cTns), gained attention in the early 1990s for the diagnosis and prognostic evaluation of patients with acute myocardial infarction (AMI). Today, cTns are considered fundamental for the management of acute coronary syndromes (ACSs) and constitute a critical step for their definition together with clinical and electrocardiographic criteria. In addition to their high accuracy for infarction detection, the use of these markers has brought great utility for the choice of different therapeutic strategies.^(^^1-6^^)^ Both substantial increases in the analytical performance and enhanced understanding of the kinetics of these substances in the presence of myocardial damage have wide applicability for cTns inside and outside of cardiology. These molecules, called high-sensitive or high-sensitivity cTnT (hsTnT) and cTnI (hsTnI), are detected earlier and at extremely low levels, thereby reducing the "blind" detection interval of fourth-generation cTn assays and aiding not only in diagnostic confirmation^(^^7^^)^ but also in detecting patients at high risk for cardiovascular events.^(^^8^^)^ According to the guidelines for non-ST-segment elevation AMI (NSTEMI) and unstable angina, these tests have resulted in a 20% relative increase in type 1 AMI detection (due to spontaneous atherosclerotic plaque fissuring and dissection) and a reduction in cases of unstable angina. However, these characteristics should be treated with caution, since these markers are present in myocardial damage of diverse etiologies, sometimes without the involvement of clinically evident myocardial necrosis (Table 1). An evaluation of the clinical context and complementary tests are extremely important, as recommended by guidelines for the correct diagnosis and definition of the therapeutic strategy to be adopted.^(^^9^^)^

**Table 1:** Reasons for cardiac troponin elevation

**Coronary causes**
Acute coronary syndromes
**Noncoronary causes**
Severely decompensated heart failure
Pulmonary embolism
Aortic dissection
Tachyarrhythmias/bradyarrhythmias
Perimyocarditis
Infective endocarditis
Takotsubo
Radiofrequency ablation
Heart contusion
**Extracardiac causes**
Shock/hypotension
Renal failure
Stroke
Strenuous physical activity
Sympathomimetic drugs
Sepsis
Chemotherapy

### Elevations in cardiac troponins during intensive therapy

**Figure 1:**
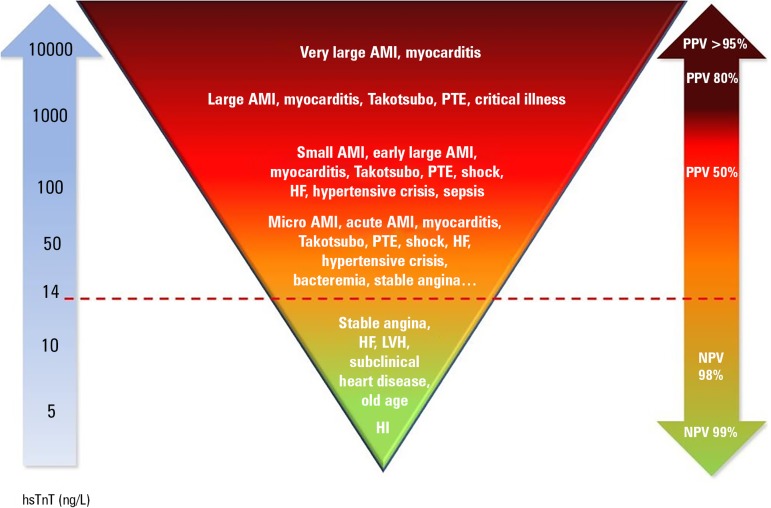
Correlation between the level of high-sensitive cardiac troponin T and diseases that cause its elevation as well as the negative and positive predictive values for the diagnosis of acute coronary syndromes. hsTnT - high-sensitive cardiac troponin T; AMI - acute myocardial infarction; PTE - pulmonary thromboembolism; HF - heart failure; LVH - left ventricular hypertrophy; HI - healthy individual; PPV - positive predictive value; NPV - negative predictive value.

Source: Adapted from Garg P, Morris P, Fazlanie AL, Vijayan S, Dancso B, Dastidar AG, et al. Cardiac biomarkers of acute coronary syndrome: from history to high-sensitivity cardiac troponin. Intern Emerg Med. 2017;12(2):147-55. Figure 3, High-sensitivity cardiac troponin as a quantitative marker; p. 150.^(^^15^^)^

The document created by the European Society of Cardiology (ESC), American College of Cardiology Foundation (ACCF), American Heart Association (AHA), and World Heart Federation (WHF) for the universal definition of AMI considers an elevation and/or drop in cTns according to the associated clinical situation to be essential for diagnostic purposes.^(^^10^^)^ According to the guideline, the criterion used for AMI is the demonstration of cTn levels above the 99^th^ percentile or a drop in the marker and at least one criterion involving clinical or complementary signs, such as electrocardiographic changes indicative of new ischemia (ST-segment depression, new or presumed new left bundle branch block, and pathological Q waves) and the demonstration of a new infarct area or segmental contractility disorders in imaging examinations. After percutaneous coronary intervention and myocardial revascularization, values three times the upper reference limit (URL) in the 99^th^ percentile are accepted. Based on the clinical circumstances, spontaneous AMI due to atherosclerotic plaque rupture, erosion, fissuring, or dissection is classified as type 1, AMI due to increased oxygen demand of the myocardium is type 2, AMI related to sudden cardiac death is type 3, AMI associated with a percutaneous procedure is type 4a, AMI associated with intracoronary stent thrombosis is type 4b, and AMI associated with myocardial revascularization surgery is type 5. The high-sensitive test also has the utility of excluding the AMI diagnosis at the initial presentation, since the negative predictive value of the test is 97 to 99%.^(^^7^^)^ With new tests, elevations are more commonly found in patients with structural heart disease, including those with obstructive coronary disease, kidney disease, and stable angina.^(^^11^^)^ In situations of stable angina, values in the 99^th^ percentile have been found in 37% of cases with plaques considered vulnerable and in up to 2% of the general population in other studies.^(^^11-14^^)^ The latter population may have heart failure, renal failure, or left ventricular hypertrophy in addition to coronary disease. In patients with compensated heart failure, hsTnT can also be found very close to the clinical decision limit (14ng/L).^(^^13^^)^ The clinical context should be considered for the correct interpretation of these markers. Thus, the management of a critical patient deserves a detailed analysis of preexisting conditions when abnormal curves from cTn assays are evaluated. The recommended flowcharts for patients with suspected ACS include a strategy that involves serial collection of the biomarker within a few hours. The diagnosis is confirmed in most cases using clinical and electrocardiographic criteria and values more than five times the URL. Serial changes are more accurate when below or near the 99^th^ percentile, and very high values and a lack of significant variation in the initial presentation are generally indicative of chronic myocardial injury, which considerably reduces their potential for the diagnosis of AMI type 1 (Figure 1).^(^^15^^)^ Each hsTnT and hsTnI assay has its specific cutoff points, and very low values from both tests can accurately rule out a clinical presentation within a few hours.^(^^9^^)^ However, diverse clinical situations and the nontypical presentation of ACS in the intensive care unit (ICU) setting impair analysis of the cTn levels combined with clinical data. Moreover, no parameter is available to evaluate the kinetics of these markers starting from the already very high values in the critical patient. Rigorous hemodynamic control, electrocardiogram, bedside echocardiography, or an imaging examination, which may demonstrate loss of viability in a new segment of the left ventricle, should be essential for the differential diagnosis and confirmation of AMI. Given elevated cTns, a chronic condition can be assumed only after the exclusion of symptoms suggestive of ischemia, a normal electrocardiogram and echocardiogram, and the absence of a rising and/or falling marker curve.

**Figure 2:**
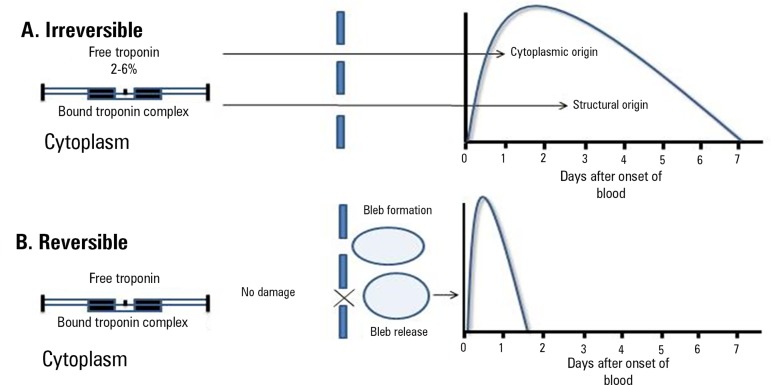
Differences between the release patterns of cardiac troponins in reversible *versus* irreversible injury.

Source: Adapted from Hickman PE, Potter JM, Aroney C, Koerbin G, Southcott E, Wu AH, et al. Cardiac troponin may be released by ischemia alone, without necrosis. Clin Chim Acta. 2010;411(5-6):318-23. Figure 3, Mechanism of troponin release; p.321.^(^^16^^)^

An important conceptual theory for the interpretation of these tests is the formation of blebs containing cTns, of which approximately 2 to 6% are free in the cytosol; these blebs release their material into the extracellular medium due to the reduced oxygen supply. When the tissue damage becomes prolonged, the blebs increase and rupture, triggering cell death. Loss of the structural content of myocardial cells is reflected in the prolonged cTn release curve in AMI. However, if reperfusion occurs or if the injury is transient, these blebs are reabsorbed or may leak their cytoplasmic contents into the circulation with the cell membrane still intact. This short detection period represents a point release of the cytoplasmic content, which corresponds to the half-life of the detected substances (Figure 2).^(^^16^^)^ Lower values found in the initial evaluation of patients for acute coronary syndrome may not be related to type 1 AMI (ischemia due to atherosclerotic plaque rupture, thrombus formation, spontaneous fissuring, or dissection), and transient elevations can be detected without clinical evidence of AMI in some situations.^(^^17^^)^ Substantial increases followed by falls should be considered for diagnosis (starting at five times the upper limit of normality) and have obtained the best positive predictive value (90%).^(^^9^^)^ This situation is observed in clinical practice and was detailed in the study by Apple et al., which demonstrated that patients with ACS had lower cTn levels and an earlier decline in the presence of unstable angina or the absence of persistent ST-segment elevation.^(^^18^^)^ Patients with persistent ST-segment elevation or progression to "Q" AMI have higher biomarker peaks and a late decline (7-10 days). The first situation refers to the predominance of ischemic cTn release and the second to necrotic release. An intermediate cTn release behavior probably exists depending on the nature of the insult, time to reperfusion therapy, clinical course, and the patient's treatment success.

### Sepsis

Sepsis and other systemic disorders may lead to myocardial depression and cell damage caused by increased consumption and/or a reduced supply of oxygen to the heart.^(^^19^^)^ Proposed mechanisms for the release of cTns in the presence of septic shock also include focal ischemia and the effect of endotoxins, cytokines, and reactive oxygen species on cardiomyocytes. Tumor necrosis factor (TNF) may modulate the activation or biosynthesis of proteases; for example, calpains and caspases may participate in the degradation of contractile proteins, including cTns.^(^^20,21^^)^

Experimental studies suggest that generalized microvascular dysfunction is a prominent sign of septic shock and may indicate relative ischemia due to the microvascular shunt effect or secondary flow heterogeneity resulting from endothelial dysfunction, capillary plugs, interstitial edema, and free radical production. Some studies suggest areas of reversible mismatch due to redistribution of the microcirculation in response to metabolic changes.^(^^22-24^^)^ Typically, cardiac output is elevated, leading to increased cardiac work and oxygen demand. The presence of tachycardia with a decreased diastolic filling time also increases oxygen consumption. The coronary reserve flow becomes limited, and ischemia may occur. Finally, treatment with high doses of inotropic agents to increase the oxygen supply may increase the incidence of cardiovascular complications and affect the outcome unfavorably in patients with adequate fluid resuscitation. Prolonged resuscitation may elevate ischemic damage with increased filling pressures, parietal strain, and additional cardiomyocyte injury during septic shock.^(^^25^^)^

Tachycardia during the hyperdynamic state is a striking finding. Stretching of the myocardial fiber and an increase in parietal tension are probable mechanisms due to the parallel increase in natriuretic peptide and cTns in several types of tachycardia.^(^^26^^)^ Although some studies have shown an association between several types of tachycardia and elevations in cTns, other factors may confound this association, such as the presence of coronary artery disease (CAD), other associated clinical conditions, and hemodynamic changes.^(^^27,28^^)^ Speculation also exists that the tachycardia present in sepsis and septic shock may cause cTn release in the absence of structural heart disease, CAD, and myocardial depressing factors (sepsis) and thus may only represent a manifestation of the imbalance between oxygen supply and demand (type 2 AMI).

Mediators, such as TNF, are also implicated in the increased permeability of the cardiomyocyte membrane. As previously reported, this phenomenon could explain the presence of cTns in the absence of irreversible cell damage.^(^^29^^)^ However, TNF values were not correlated with elevations in cTns in healthy humans tested with endotoxin, suggesting that other mechanisms must be involved. The interleukin 6 (IL-6) level also contributes to increased cell permeability; the level is elevated significantly in patients with septic shock and positive for cTns compared to the level in those negative for cTns.^(^^30^^)^ Additionally, cTn-positive patients have a higher level of histological changes. Necrosis with contraction bands (coagulative myocytolysis) and fibrillar rupture is more commonly associated with calcium overload and typically is associated with reperfusion injuries and the use of catecholaminergic drugs.^(^^31^^)^

The prognosis of sepsis depends on the severity of organ dysfunctions, especially cardiovascular dysfunction. Many studies have discussed the fact that cTns are independent parameters of the outcome, with no distinction between the causes of elevation of these biomarkers.^(^^32-34^^)^ When restricted to sepsis cases, elevated levels have been shown to be associated with the disease severity. Generally, these results are very similar to those showing increased levels indicating worse left ventricular function and a consequent unfavorable outcome.^(^^35^^)^ The study by Røsjø et al. indicated that hsTnT was an important early marker of circulatory dysfunction in what was then termed severe sepsis but was not added to the Simplified Acute Physiology Score (SAPS II) for the prediction of hospital mortality. In this situation, cardiovascular dysfunction may not be the only factor responsible for mortality in sepsis. hsTnT was present in all initial cases of severe sepsis, whereas 60% of patients had positive fourth-generation cTnT. The authors concluded that an initial hsTnT level of less than 14ng/L (99^th^ percentile) in early severe sepsis might indicate a low probability of progression to septic shock.^(^^34^^)^ A meta-analysis published in 2013 showed that both cTnT and cTnI were independent factors of mortality regardless of the causes or comorbidities involved in their elevation and corrected for confounding biases, such as renal failure or heart disease present prior to inclusion in the study.^(^^35^^)^ The field of cardiology shares the same hypothesis, because evidence suggests that mortality increases in patients admitted with hsTnT levels above the 99^th^ percentile regardless of the causal agent of cTn elevation. The SWEDEHEART registry noted that patients admitted with suspected acute coronary syndrome and hsTnT levels above 14ng/L showed higher mortality rates, although only 18.2% of the patients actually had type 1 AMI.^(^^36^^)^

### Stroke

After introduction of the sensitive myocardial necrosis markers, the association between cTns and stroke was demonstrated extensively. A systematic review of 15 studies prior to the use of high-sensitive cTns showed that 18.1% of patients had elevated levels during the event.^(^^37^^)^ With the use of the high-sensitive tests, this correlation reached 60% in some cohort analyses. When studies evaluated serial changes in the biomarker, 60% of the patients had stable cTn levels, and the rest had a 20% rise and fall pattern.^(^^38^^)^ The latter pattern may be related to a coronary event (type 1 AMI) or myocardial injury secondary to stroke. Acute changes in autonomic control with exaggerated catecholamine release may be a noncoronary consequence of increased cTns in stroke.^(^^39^^)^ The insula is an important region for autonomic regulation and is frequently affected by involvement of the anterior circulation, and an association between stroke in this cortical area and an increase in cTn values has been demonstrated.^(^^40^^)^ However, other studies have not demonstrated a correlation between the cTn values and the location or volume of the infarction.^(^^41^^)^ We add that exposure to excessive catecholaminergic stimulation and myocardial ischemia due to coronary vasoconstriction or pre-existing CAD may occur in type 2 AMI. The frequency of associated type 1 AMI is uncertain, but after adjusting for age and sex, patients with ischemic stroke are less likely to have associated coronary lesions despite elevations of the cTn levels similar to cases of NSTEMI; moreover, half of the patients in one study had no coronary lesions on the angiogram.^(^^42^^)^ Hospital mortality is higher in patients with intracranial hemorrhage than in those with ischemic stroke, and the risk associated with cTn elevation is higher in ischemic stroke than in hemorrhagic stroke.^(^^43^^)^

Thus, in patients with stroke and elevated cTns, an initial assessment of whether an acute increase in the biomarker has occurred or whether it is elevated but stable is reasonable. Then, a careful assessment whether an associated ACS exists is essential. Once a high but stable value is detected, we should look for comorbidities associated with cTn elevation that may benefit from more aggressive long-term management. A frequent situation is encountering abnormal cTn levels in stroke patients with a recent ACS or dilated cardiomyopathy complicated by atrial fibrillation preceding the event.

### Heart failure

Biomarkers in the context of acutely decompensated heart failure have become important for diagnostic and prognostic assessments and are currently part of the standard clinical evaluation. Current recommendations indicate the use of natriuretic peptides.^(^^44^^)^ However, because heart failure is a complex syndrome, single biomarker assessment may not reflect all of its characteristics. The accumulated evidence emphasizes that cTns can add information to natriuretic peptides. After that description, subsequent studies correlated cTn levels with the disease severity. The cTn levels do not allow the etiology to be diagnosed but may reflect the increase in the left ventricular mass and provide data on the ejection fraction and diastolic dysfunction.^(^^45^^)^ Elevation in the cTn levels was associated with the symptom severity, increased need for supportive therapy with vasopressors and inotropes, and worsening of the outcome at 30 days.^(^^44^^)^

A meta-analysis based on 77,297 patients concluded that detecting cTns in heart failure patients increased their 1-year mortality and readmission rates, demonstrating a hazard ratio of 2.3 (95%CI 1.8 - 3.0).^(^^46^^)^ These findings also apply to high-sensitive tests. Serial changes in the cTn concentrations are also important, because the persistence of elevated cTn levels during or after hospitalization is related to worse outcomes, whereas decreasing or stable values are associated with lower adverse outcome rates.^(^^47^^)^ Despite this finding, the clinical usefulness is uncertain. The challenge persists in the differentiation between ACS-mediated release and noncoronary release. A Class I recommendation with Level of Evidence C is included in the European guidelines for the use of cTns in acute heart failure.^(^^44^^)^ However, the cTn concentrations should be interpreted broadly. A careful evaluation with imaging and coronary angiography should be part of the investigation, especially in cases of recent-onset heart failure, positive cTns, and high clinical suspicion of ischemia of coronary origin.

### Pericarditis and myocarditis

Epicardial involvement may be a striking finding in acute pericarditis, and cTns are elevated in approximately 30 to 49% of cases. When they reflect myocardial injury, the finding of cTns together with other findings compatible with global or regional left ventricular dysfunction may indicate myopericarditis.

Elevation of the biomarker does not seem to be related to the prognosis of myopericarditis and is considered a weak marker of the extent of myocardial involvement.^(^^19^^)^

Although they appear to be useful for myocarditis diagnosis, cTns have limited sensitivity, at least in studies using less sensitive markers. Better accuracy was gained with hsTnT in one study (area under the curve - AUC: 0.878; p = 0.002) with a sensitivity of 83% and specificity of 80% achieved using a cutoff of 50ng/L.^(^^48^^)^ The marker concentrations in that study showed different behavior according to the clinical presentation. Patients presenting symptoms for more than 13 days had substantially lower values. In contrast to other clinical conditions, cTn concentrations in myocarditis do not imply an adverse prognosis. Increased cTn levels were not useful to predict complications during follow-up of 486 patients with acute pericarditis or myopericarditis, and a systematic review including eight studies concluded that cTns were not able to predict adverse events during clinical follow-up.^(^^49,50^^)^ Nevertheless, the reduced rate of events and the small sample size in these studies should be considered.

### Takotsubo cardiomyopathy

Takotsubo cardiomyopathy, which is also called stress-induced cardiomyopathy or apical ballooning syndrome, is reported in approximately 0.7 to 2.5% of suspected ACS cases.^(^^51^^)^

Although we observe Takotsubo cardiomyopathy most commonly in women with emotional or physiological stress with a presentation of cardiomyopathy, it may occur in a broader clinical spectrum, including presentation in younger men and in those without a triggering event.^(^^52^^)^

Most patients have a mild to moderate cTn increase within 24 hours of presentation.^(^^53^^)^ This elevation is disproportionate to the finding of regional left ventricular dysfunction on imaging tests.^(^^54^^)^

Some studies have attempted to differentiate patients with Takotsubo cardiomyopathy from those with ACS using biomarker behavior, since diagnostic confirmation is often performed only after finding no obstructive lesions on coronary angiography and normal ventriculography. A prospective analysis of the magnitude of the elevations in cTnT and cTnI found that cases with the former below 6ng/mL and the latter below 15ng/mL showed little probability of being Takotsubo cardiomyopathy.

Because the presentation more closely involves regional left ventricular changes than loss of viability due to necrosis, the ratio between markers has been studied at ACS patient admission. The ratio that best differentiated Takotsubo and ST-segment elevation AMI (STEMI) was the ratio between peaks of the N-terminal prohormone of brain natriuretic peptide (NT-proBNP) in ng/L to cTnT in µg/L. A cutoff value of 2,889 was able to distinguish Takotsubo cardiomyopathy and STEMI (91% sensitivity and 95% specificity), and a ratio of 5,000 was able to discriminate Takotsubo cardiomyopathy and NSTEMI (83% sensitivity and 95% specificity).^(^^55^^)^

### Myocardial contusion

The incidence of myocardial contusion in patients with closed chest trauma ranges from 3% to 56% of cases depending on the diagnostic criteria used. The lack of specific signs and symptoms and the broad spectrum of clinical presentation make its evaluation difficult.^(^^56^^)^

Both cTnT and cTnI have an equivalent sensitivity profile and greater accuracy for myocardial contusion detection. These indicators aid in the selection of patients who must remain under intensive cardiac monitoring.^(^^57^^)^

A study identified that patients with brain injury, thoracic injury, closed chest trauma, and shock more frequently had higher cTnI values. In the same analysis, sustained (greater than 36 hours) and significant cTnI release (cTnI peak ≥ 2µg/L) was more frequently associated with thoracic trauma (82%) and the presence of electrocardiographic changes. Based on electrocardiographic abnormalities, the sensitivity, specificity, and positive and negative predictive values of cTnI release were 63%, 98%, 40%, and 98%, respectively. Mortality could not be discriminated according to the marker values, but patients with a normal electrocardiogram and cTnI level 8 hours after chest trauma had an almost null probability of complicated myocardial contusion and required no additional evaluation.^(^^58^^)^ Patients with ECG changes, elevated cTn, or both must remain under observation in the ICU for at least 24 hours, during which time most complications of myocardial contusion develop.^(^^56^^)^

### Pulmonary thromboembolism

Pulmonary thromboembolism patients with signs of shock and hypotension present high mortality. After careful risk assessment, the use of thrombolytics in these patients is generally accepted.^(^^59^^)^ Intermediate-risk patients are considered those with signs of right ventricular dysfunction who are hemodynamically stable and cTn positive. Kucher et al. concluded that a normal echocardiogram and negative cTns were useful for identifying patients with a lower risk of early mortality.^(^^60^^)^ However, the reason for the increase in these markers in pulmonary thromboembolism is still unclear. In one study, 63% of cases with right ventricular dilatation had elevated cTns, whereas 29% of cases with positive cTns had a normal end-diastolic diameter.^(^^61^^)^ cTnI was also associated with more segmental defects on ventilation/perfusion scintigraphy. However, hypoxia secondary to the decrease in the ventilation/perfusion ratio, hypoperfusion due to shock and coronary flow decrease, and systemic vein to coronary artery embolism by patent foramen ovale can also be considered origins of the cTn elevation. Transmural right ventricular infarction despite normal coronary arteries has also been found in cases of massive pulmonary thromboembolism.^(^^62^^)^ When evaluating kinetics, some studies have concluded that the cTnT peak is lower and shorter than that of AMI.^(^^63^^)^ Although this behavior suggests that cTns are released from the cytosolic pool during ischemia, given such release kinetics, this hypothesis still requires testing. A meta-analysis of 20 studies with 1,985 participants demonstrated that cTn elevation was significantly associated with increased short-term mortality resulting from thromboembolism, including situations with a preserved hemodynamic status.^(^^64^^)^ Another recent meta-analysis of 1,366 patients identified a four-fold greater risk of short-term death in 55 patients.^(^^65^^)^

### Advanced or terminal renal disease

The pathophysiological mechanism by which the cTn levels are elevated in chronic renal failure (CRF) remains uncertain. The most investigated associations are the presence of diffuse obstructive CAD with microinfarcts and left ventricular hypertrophy. A strong association exists between the cTnT level and the presence of multivessel CAD in patients with asymptomatic CRF undergoing coronary angiography.^(^^10^^)^

A study underscored the challenges in interpreting AMI in CRF using the 99^th^ percentile derived from the healthy general population as a cutoff. In contrast to patients with preserved renal function, those with CRF more commonly have baseline cTn above the 99^th^ percentile, particularly when the assay is highly sensitive.^(^^66^^)^ Therefore, a serial cTn analysis with a relative increase of approximately 20%, as recommended by the guidelines, should be performed in the appropriate clinical context for the diagnosis of AMI.^(^^9^^)^ Both cTnT and cTnI can be used, and there is no consensus in the guidelines on the advantages of cTnI over cTnT for AMI diagnosis in patients with CRF.^(^^10^^)^ Importantly, both cTns are markers of diagnostic choice, and no substitutes are acceptable, such as the MB fraction of creatine kinase, with the prerogative of loss of cTn specificity in this context.

A study suggested that URL elevation occurred in this group of patients. In an analysis of 75 patients with CRF, hsTnT was used with high accuracy for the diagnosis of AMI at a level twice the 99^th^ percentile of the URL, resulting in a sensitivity of 94% and specificity of 86%. However, this change may adversely change the sensitivity of the assay, and use of the URL provided by the manufacturer and/or institution for the exclusion or confirmation of AMI is advisable in these cases.^(^^11^^)^ The diagnostic accuracy of high-sensitivity assays is also compromised in hemodialysis patients, since almost all patients have baseline levels above the 99^th^ percentile. In a series of 670 hemodialysis patients evaluated for dyspnea or chest pain, the receiver operating characteristic (ROC) curve for the hsTnT test was only 0.68 but significantly improved to 0.9 with serial evaluation over 3 hours. The most favorable cutoff point for the relative variation was 24%.^(^^67^^)^ However, clinical judgment is a critical component for assessing chest pain in hemodialysis patients. Although the dynamic changes in markers improve the specificity for the diagnosis of AMI in patients with CRF, relying on this parameter alone may be associated with a loss of up to 12% of STEMI cases.^(^^68^^)^

During evaluation of a patient with CRF, a baseline cTnT or cTnI value should never be interpreted as part of the loss in glomerular filtration alone. Even without the association with AMI, elevated levels indicate a worse prognosis and should be valued.^(^^69,70^^)^

### Strenuous physical exercise

Cardiac troponins may be elevated immediately after strenuous physical exercise, which is a phenomenon that has been studied in long-distance runners.^(^^71^^)^ The involvement of the cardiac musculature manifests transiently with falls in the systolic and diastolic functions; this phenomenon is called "heart fatigue".^(^^72^^)^

High-sensitive cTns are detected in approximately 80-86% of marathon runners after exercise.^(^^73^^)^ In fact, these assays may be elevated during short periods of exercise in both cardiopathic and noncardiopathic patients.^(^^17,74^^)^ Several studies have demonstrated that transient elevations do not indicate myocardial damage, since the levels normalize within 24 to 48 hours.^(^^19^^)^ These data also reinforce the theory of cTn release in the cytosol and not from the structural content of the myocytes.

In a meta-analysis of 1,120 individuals, Shave et al. found that the exercise duration was related to increased cTnT levels without correlation with the participants' ages. With the use of high-sensitive assays, a negative correlation was also found between the cTn levels and performance, and a nonsignificant correlation was found with age. This finding can help the clinician evaluate symptomatic patients after competitive exercises in practice.^(^^75^^)^

Interestingly, transient alterations in right ventricular function were found in high-intensity athletes. However, this finding has not added prognostic information to date.^(^^76^^)^

The clinical impact of exercise-induced cTn elevations is still unclear. Symptoms during exercise are relatively common, and marathoners presenting with dizziness, chest pain, and sometimes circulatory collapse may pose a challenge for diagnosis in the presence of elevated cTns. Currently, there are no data to discourage athletes from competitive activity due to elevation in cTns after exertion. Moreover, D-dimers and natriuretic peptides as well as cTns may be elevated after exercise.^(^^19^^)^

Lack of attention to compatible findings after high-intensity efforts, including biomarker elevations and transient right ventricular dysfunction in the absence of clinical signs indicative of ACS, heart failure, or pulmonary embolism, can trigger a costly, invasive, and unnecessary investigation. Treating the patient and not the clinical assay is still a fundamental precept.

### Type 1 and type 2 acute myocardial infarction and myocardial injury

Since the last AMI definition, diagnostic choice markers have emerged to support clinical and electrocardiographic data. However, one of the major challenges in the ICU environment is the differentiation between increases in cTns due to hemodynamic instability, sepsis, and other disorders and type 1 AMI. Electrocardiographic abnormalities may not provide diagnostic help, since the presence of ST-segment elevation can occur in cases of type 2 AMI. Spatz et al. found that ST-segment elevation was present in 17% or 15% of type 2 AMI cases where obstructive CAD was present or absent, respectively.^(^^77^^)^ Similarly, another study reported that ST-segment elevation was present in 16% of obstructive CAD and 11% of nonobstructive CAD cases, respectively.^(^^78^^)^ The recommendation is that coronary angiography should be used as an investigation if no unequivocal factor is the cause of type 2 AMI, such as known hemorrhagic shock in CAD.^(^^79^^)^

**Figure 3:**
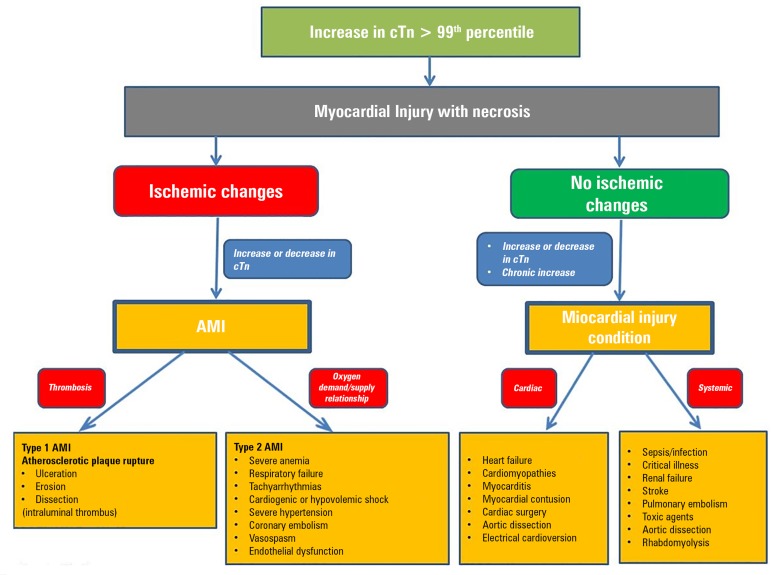
Model of differentiation between acute myocardial infarction and myocardial injury. cTn - cardiac troponin; AMI - acute myocardial infarction.

Source: Adapted from Sandoval Y, Thygesen K. Myocardial infarction type 2 and myocardial injury. Clin Chem. 2017;63(1):101-7.^(^^79^^)^

The absence of spontaneous rupture and dissection of atherosclerotic plaques is a purely clinical analysis and increases the ambiguity of the AMI diagnosis due to imbalance between oxygen supply and demand, contributing to disparities in its incidence (1.6% to 26%). The highest incidence was obtained with more restrictive criteria. In addition, several studies did not place supply or demand thresholds for oxygen, since several anatomical and pathophysiological factors could be blamed. Patients with sepsis are also sometimes included in analyses with those with type 2 AMI. Sepsis and septic shock share other factors besides the imbalance in oxygen demand and supply in their pathophysiology. Type 2 AMI is diagnosed when there is evidence of necrosis due to increased and decreased cTns with at least one value above the 99^th^ percentile, in a clinical situation consistent with myocardial ischemia, and in cases with an evident imbalance between oxygen supply and demand without atherosclerotic plaque rupture and with one more criterion according to the universal definition. Figure 3 shows a conceptual model of differentiation between myocardial injury and AMI.^(^^79^^)^

### Management and prognosis of type 2 acute myocardial infarction

Since the term was created in 2007, no practical guidelines have been available for management of type 2 AMI. Observational studies show that such patients are managed less frequently with revascularization, beta-blockers, angiotensin-converting enzyme inhibitors, statins, and antiplatelet agents, even when significant CAD is present.^(^^79^^)^

Shah et al. examined the impact of lowering the threshold of a high-sensitive troponin test based on the incidence, management, and outcomes of type 1 and type 2 AMI and myocardial injury. These tests resulted in higher costs, greater use of hospital resources, and a better prognosis for type 1 AMI.^(^^80^^)^ However, for patients with type 2 AMI, there was an increase in indications for specialized cardiovascular evaluation, echocardiograms, and coronary angiography, which had no impact on the treatment or prognosis. No specific therapy was available in these cases, and a missed opportunity actually existed. In the acute phase, it is intuitive to carry out strategies for adequate oxygen supply and reduced oxygen demand, including volemic state adjustment, pressure, and inotropic support, if necessary; the use of blood products; heart rate control; and ventilatory support with an active search for the causal factor. Depending on the clinical situation, coronary angiography should be used to investigate the presence of CAD. If applicable, the guidelines should be followed. However, if obstructive CAD is absent, strategies for risk reduction are scarce. In general, despite the different definitions used among studies, there is a very somber prognosis for type 2 AMI, with long-term mortality reaching 63% after 3 years of follow-up.^(^^78^^)^ Studies have focused on all-cause mortality at the expense of cardiovascular causes.

## CONCLUSION

With the advent of high-sensitive troponin assays, both the detection sensitivity of acute myocardial infarction and the detection of elevated marker levels in situations unrelated to acute coronary syndromes have increased. The increase in the prevalence of noncoronary conditions, which are associated with increases in cardiac troponins, presents challenges for the diagnosis of acute myocardial infarction, especially in the elderly population, in which coronary and noncoronary heart diseases are found. Several strategies for the use of these markers have been studied without a definitive opinion in the current guidelines. Considering elevated cardiac troponin values at the initial presentation is important, since the predictive value of the test increases for cardiovascular and noncardiovascular diseases. The behavior of the serial test can also help identify several situations, including acute myocardial infarction. This evaluation, combined with a rigorous clinical analysis, should increase the use of earlier therapeutic strategies and exclude patients with greater safety.
